# Ethyl 5-bromo-1-benzofuran-2-carboxyl­ate

**DOI:** 10.1107/S1600536811005897

**Published:** 2011-02-23

**Authors:** Hatem A. Abdel-Aziz, Ahmed Bari, Seik Weng Ng

**Affiliations:** a Department of Pharmaceutical Chemistry, College of Pharmacy, King Saud University, Riyadh 11451, Saudi Arabia; b Department of Chemistry, University of Malaya, 50603 Kuala Lumpur, Malaysia

## Abstract

In the title compound, C_11_H_9_BrO_3_, the benzofuran fused-ring system is almost planar, with a maximum atomic deviation of 0.024 (5) Å; the carboxyl –CO_2_ fragment is aligned at 4.8 (7)° with respect to the fused-ring plane. Weak inter­molecular C—H⋯O hydrogen bonding is present in the crystal structure. π–π stacking is also observed between parallel mol­ecules, the centroid–centroid distance between benzene and furan rings of adjacent mol­ecules being 3.662 (3) Å.

## Related literature

For our previous reports of the pharmacological properties of benzofurans, see: Abdel-Aziz & Mekawey (2009 ▶); Abdel-Aziz *et al.* (2009 ▶). For a related structure, see: Kossakowski *et al.* (2005 ▶).
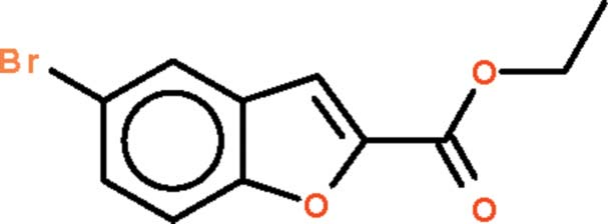


## Experimental

### 

#### Crystal data

C_11_H_9_BrO_3_
*M* *_r_* = 269.09
Monoclinic, 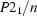

*a* = 3.8869 (3) Å
*b* = 23.780 (2) Å
*c* = 11.0820 (7) Å
β = 96.905 (8)°
*V* = 1016.89 (13) Å^3^
*Z* = 4
Mo *K*α radiation
μ = 4.02 mm^−1^
*T* = 100 K
0.30 × 0.20 × 0.10 mm

#### Data collection

Agilent SuperNova Dual diffractometer with an Atlas detector
Absorption correction: multi-scan (*CrysAlis PRO*; Agilent, 2010 ▶) *T*_min_ = 0.378, *T*_max_ = 0.689
6060 measured reflections
2250 independent reflections
1843 reflections with *I* > 2σ(*I*)
*R*_int_ = 0.045

#### Refinement

*R*[*F* ^2^ > 2σ(*F* ^2^)] = 0.055
*wR*(*F* ^2^) = 0.109
*S* = 1.18
2250 reflections
136 parameters
H-atom parameters constrained
Δρ_max_ = 0.97 e Å^−3^
Δρ_min_ = −0.71 e Å^−3^

### 

Data collection: *CrysAlis PRO* (Agilent, 2010 ▶); cell refinement: *CrysAlis PRO*; data reduction: *CrysAlis PRO*; program(s) used to solve structure: *SHELXS97* (Sheldrick, 2008 ▶); program(s) used to refine structure: *SHELXL97* (Sheldrick, 2008 ▶); molecular graphics: *X-SEED* (Barbour, 2001 ▶); software used to prepare material for publication: *publCIF* (Westrip, 2010 ▶).

## Supplementary Material

Crystal structure: contains datablocks global, I. DOI: 10.1107/S1600536811005897/xu5164sup1.cif

Structure factors: contains datablocks I. DOI: 10.1107/S1600536811005897/xu5164Isup2.hkl

Additional supplementary materials:  crystallographic information; 3D view; checkCIF report

## Figures and Tables

**Table 1:** Hydrogen-bond geometry (Å, °)

*D*—H⋯*A*	*D*—H	H⋯*A*	*D*⋯*A*	*D*—H⋯*A*
C2—H2⋯O2^i^	0.95	2.57	3.400 (6)	146
C11—H11*A*⋯O2^ii^	0.98	2.53	3.472 (6)	160

Symmetry codes: (i) 
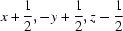
; (ii) 
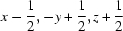
.

**Fig. 1.**
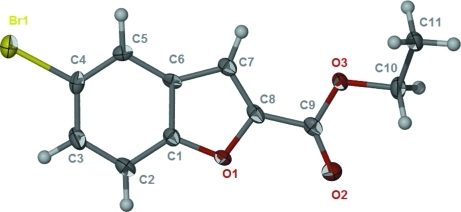
Thermal ellipsoid plot (Barbour, 2001) of C11H9BrO3 at the 70% probability level; hydrogen atoms are drawn as spheres of arbitrary radius.

## supplementary crystallographic information

### Comment

Ethyl 5-bromobenzofuran-2-carboxylate (Scheme I) is a commercially available chemical that has been evaluated for its pharmacological properties. We have reported the pharmacological properties of related compounds (Abdel-Aziz & Mekawey, 2009; Abdel-Aziz *et al.*, 2009). The title compound is an approximately planar molecule; the carboxyl –CO_2_ fragment is aligned at 4.8 (7)° with respect to the benzofuran fused-ring (Fig. 1). Bond dimensions are similar to those found in methyl 7-methoxybenzofuran-2-carboxylate (Kossakowski *et al.*, 2005).

### Experimental

5-Bromosalicyladehyde (2.01 g, 10 mm l), diethyl bromomalonate (2.63 g 11 mmol) and potassium carbonate (2.28 g, 20 mmol) were heated in 2-butanone (20 ml) for 14 h. The solvent was evaporated and water was added to the residue. The organic compound was extracted by ether. The ether phase was washed with 5% sodium hydroxide. The ether was then evaporated and the product recrystallized from ethanol to give the title ester, m.p. 333–335 K.

### Refinement

Carbon-bound H-atoms were placed in calculated positions [C—H 0.95 to 0.98 Å, *U*_iso_(H) 1.2 to 1.5*U*_eq_(C)] and were included in the refinement in the riding model approximation.

### Crystal data

**Table:**

C_11_H_9_BrO_3_	*F*(000) = 536
*M**_r_* = 269.09	*D*_x_ = 1.758 Mg m^−^^3^
Monoclinic, *P*2_1_/*n*	Mo *K*α radiation, λ = 0.71073 Å
Hall symbol: -P 2yn	Cell parameters from 2599 reflections
*a* = 3.8869 (3) Å	θ = 2.5–29.3°
*b* = 23.780 (2) Å	µ = 4.02 mm^−^^1^
*c* = 11.0820 (7) Å	*T* = 100 K
β = 96.905 (8)°	Prism, colorless
*V* = 1016.89 (13) Å^3^	0.30 × 0.20 × 0.10 mm
*Z* = 4	

### Data collection

**Table:**

Agilent SuperNova Dual diffractometer with an Atlas detector	2250 independent reflections
Radiation source: SuperNova (Mo) X-ray Source	1843 reflections with *I* > 2σ(*I*)
Mirror	*R*_int_ = 0.045
Detector resolution: 10.4041 pixels mm^-1^	θ_max_ = 27.5°, θ_min_ = 2.5°
ω scans	*h* = −3→5
Absorption correction: multi-scan (*CrysAlis PRO*; Agilent, 2010)	*k* = −30→30
*T*_min_ = 0.378, *T*_max_ = 0.689	*l* = −13→14
6060 measured reflections	

### Refinement

**Table:**

Refinement on *F*^2^	Primary atom site location: structure-invariant direct methods
Least-squares matrix: full	Secondary atom site location: difference Fourier map
*R*[*F*^2^ > 2σ(*F*^2^)] = 0.055	Hydrogen site location: inferred from neighbouring sites
*wR*(*F*^2^) = 0.109	H-atom parameters constrained
*S* = 1.18	*w* = 1/[σ^2^(*F*_o_^2^) + (0.0086*P*)^2^ + 5.1797*P*] where *P* = (*F*_o_^2^ + 2*F*_c_^2^)/3
2250 reflections	(Δ/σ)_max_ = 0.001
136 parameters	Δρ_max_ = 0.97 e Å^−^^3^
0 restraints	Δρ_min_ = −0.71 e Å^−^^3^

### Fractional atomic coordinates and isotropic or equivalent isotropic displacement parameters (Å^2^)

**Table:**

	*x*	*y*	*z*	*U*_iso_*/*U*_eq_	
Br1	0.84947 (14)	0.52531 (2)	0.18162 (5)	0.02119 (16)	
O1	0.2581 (9)	0.32380 (14)	0.4040 (3)	0.0157 (7)	
O2	0.0007 (10)	0.25870 (14)	0.5783 (3)	0.0228 (9)	
O3	0.1752 (9)	0.32488 (14)	0.7194 (3)	0.0165 (8)	
C1	0.3994 (13)	0.3662 (2)	0.3427 (4)	0.0146 (10)	
C2	0.4194 (14)	0.3670 (2)	0.2183 (4)	0.0189 (11)	
H2	0.3416	0.3363	0.1672	0.023*	
C3	0.5588 (15)	0.4150 (2)	0.1733 (4)	0.0216 (12)	
H3	0.5746	0.4181	0.0886	0.026*	
C4	0.6767 (13)	0.4590 (2)	0.2510 (4)	0.0163 (11)	
C5	0.6666 (13)	0.4576 (2)	0.3740 (4)	0.0151 (10)	
H5	0.7541	0.4878	0.4248	0.018*	
C6	0.5205 (12)	0.4095 (2)	0.4220 (4)	0.0135 (10)	
C7	0.4517 (13)	0.3916 (2)	0.5397 (4)	0.0153 (10)	
H7	0.5060	0.4113	0.6141	0.018*	
C8	0.2932 (14)	0.3408 (2)	0.5243 (4)	0.0161 (11)	
C9	0.1403 (13)	0.3029 (2)	0.6073 (4)	0.0157 (10)	
C10	0.0273 (14)	0.2924 (2)	0.8131 (4)	0.0202 (11)	
H10A	0.1957	0.2639	0.8488	0.024*	
H10B	−0.1857	0.2728	0.7775	0.024*	
C11	−0.0555 (14)	0.3333 (2)	0.9091 (4)	0.0202 (11)	
H11A	−0.1540	0.3130	0.9738	0.030*	
H11B	−0.2234	0.3611	0.8728	0.030*	
H11C	0.1571	0.3526	0.9433	0.030*	

### Atomic displacement parameters (Å^2^)

**Table:**

	*U*^11^	*U*^22^	*U*^33^	*U*^12^	*U*^13^	*U*^23^
Br1	0.0225 (3)	0.0210 (3)	0.0205 (3)	−0.0006 (2)	0.0046 (2)	0.0051 (2)
O1	0.020 (2)	0.0151 (17)	0.0107 (15)	−0.0015 (15)	−0.0019 (14)	−0.0010 (14)
O2	0.032 (2)	0.0169 (19)	0.0191 (18)	−0.0038 (17)	0.0022 (17)	−0.0014 (15)
O3	0.019 (2)	0.0196 (18)	0.0108 (15)	−0.0032 (15)	0.0007 (14)	0.0015 (14)
C1	0.015 (3)	0.016 (2)	0.013 (2)	0.003 (2)	−0.0009 (19)	0.0018 (19)
C2	0.021 (3)	0.022 (3)	0.013 (2)	0.002 (2)	−0.001 (2)	−0.002 (2)
C3	0.032 (3)	0.021 (3)	0.013 (2)	0.004 (2)	0.006 (2)	0.002 (2)
C4	0.013 (3)	0.018 (2)	0.019 (2)	0.002 (2)	0.005 (2)	0.007 (2)
C5	0.013 (3)	0.014 (2)	0.018 (2)	0.000 (2)	0.001 (2)	−0.003 (2)
C6	0.008 (2)	0.018 (2)	0.012 (2)	0.001 (2)	−0.0063 (19)	−0.0007 (19)
C7	0.016 (3)	0.016 (2)	0.013 (2)	0.004 (2)	−0.001 (2)	−0.0006 (19)
C8	0.021 (3)	0.016 (2)	0.010 (2)	0.007 (2)	0.000 (2)	0.0000 (19)
C9	0.014 (3)	0.019 (3)	0.013 (2)	0.005 (2)	−0.003 (2)	0.001 (2)
C10	0.023 (3)	0.022 (3)	0.016 (2)	−0.003 (2)	0.004 (2)	0.006 (2)
C11	0.020 (3)	0.027 (3)	0.014 (2)	−0.007 (2)	0.001 (2)	0.003 (2)

### Geometric parameters (Å, °)

**Table:**

Br1—C4	1.912 (5)	C5—C6	1.410 (7)
O1—C1	1.367 (6)	C5—H5	0.9500
O1—C8	1.384 (5)	C6—C7	1.428 (6)
O2—C9	1.208 (6)	C7—C8	1.357 (7)
O3—C9	1.340 (5)	C7—H7	0.9500
O3—C10	1.465 (6)	C8—C9	1.464 (7)
C1—C2	1.390 (6)	C10—C11	1.505 (7)
C1—C6	1.398 (7)	C10—H10A	0.9900
C2—C3	1.382 (7)	C10—H10B	0.9900
C2—H2	0.9500	C11—H11A	0.9800
C3—C4	1.397 (7)	C11—H11B	0.9800
C3—H3	0.9500	C11—H11C	0.9800
C4—C5	1.369 (6)		
			
C1—O1—C8	105.3 (4)	C8—C7—H7	126.8
C9—O3—C10	116.5 (4)	C6—C7—H7	126.8
O1—C1—C2	125.2 (4)	C7—C8—O1	111.9 (4)
O1—C1—C6	110.8 (4)	C7—C8—C9	133.0 (4)
C2—C1—C6	124.0 (5)	O1—C8—C9	115.1 (4)
C3—C2—C1	116.1 (5)	O2—C9—O3	125.3 (5)
C3—C2—H2	121.9	O2—C9—C8	124.9 (4)
C1—C2—H2	121.9	O3—C9—C8	109.8 (4)
C2—C3—C4	120.6 (4)	O3—C10—C11	107.2 (4)
C2—C3—H3	119.7	O3—C10—H10A	110.3
C4—C3—H3	119.7	C11—C10—H10A	110.3
C5—C4—C3	123.4 (5)	O3—C10—H10B	110.3
C5—C4—Br1	118.2 (4)	C11—C10—H10B	110.3
C3—C4—Br1	118.4 (4)	H10A—C10—H10B	108.5
C4—C5—C6	117.1 (4)	C10—C11—H11A	109.5
C4—C5—H5	121.5	C10—C11—H11B	109.5
C6—C5—H5	121.5	H11A—C11—H11B	109.5
C1—C6—C5	118.8 (4)	C10—C11—H11C	109.5
C1—C6—C7	105.6 (4)	H11A—C11—H11C	109.5
C5—C6—C7	135.6 (5)	H11B—C11—H11C	109.5
C8—C7—C6	106.4 (4)		
			
C8—O1—C1—C2	−179.8 (5)	C4—C5—C6—C7	−178.1 (5)
C8—O1—C1—C6	−0.3 (5)	C1—C6—C7—C8	−1.0 (6)
O1—C1—C2—C3	177.3 (5)	C5—C6—C7—C8	177.8 (6)
C6—C1—C2—C3	−2.1 (8)	C6—C7—C8—O1	0.9 (6)
C1—C2—C3—C4	1.2 (8)	C6—C7—C8—C9	−175.5 (5)
C2—C3—C4—C5	0.7 (8)	C1—O1—C8—C7	−0.4 (6)
C2—C3—C4—Br1	−177.6 (4)	C1—O1—C8—C9	176.7 (4)
C3—C4—C5—C6	−1.6 (7)	C10—O3—C9—O2	−0.7 (7)
Br1—C4—C5—C6	176.6 (4)	C10—O3—C9—C8	178.6 (4)
O1—C1—C6—C5	−178.3 (4)	C7—C8—C9—O2	178.6 (6)
C2—C1—C6—C5	1.2 (8)	O1—C8—C9—O2	2.3 (7)
O1—C1—C6—C7	0.8 (5)	C7—C8—C9—O3	−0.7 (8)
C2—C1—C6—C7	−179.7 (5)	O1—C8—C9—O3	−177.1 (4)
C4—C5—C6—C1	0.7 (7)	C9—O3—C10—C11	−154.2 (4)

### Hydrogen-bond geometry (Å, °)

**Table:**

*D*—H···*A*	*D*—H	H···*A*	*D*···*A*	*D*—H···*A*
C2—H2···O2^i^	0.95	2.57	3.400 (6)	146
C11—H11A···O2^ii^	0.98	2.53	3.472 (6)	160

Symmetry codes: (i) *x*+1/2, −*y*+1/2, *z*−1/2; (ii) *x*−1/2, −*y*+1/2, *z*+1/2.
